# Probiotic Supplementation Improves Hematological Indices and Morphology of Red Blood Cells and Platelets in Obese Women: A Double-Blind, Controlled Pilot Study

**DOI:** 10.3390/metabo15050310

**Published:** 2025-05-06

**Authors:** Nina Okuka, Nevena Dj. Ivanovic, Neda Milinkovic, Snezana Polovina, Mirjana Sumarac-Dumanovic, Rajna Minic, Brizita Djordjevic, Ksenija Velickovic

**Affiliations:** 1 Department of Bromatology, Faculty of Medicine, University of Banja Luka, 78000 Banja Luka, Bosnia and Herzegovina; nina.okuka@med.unibl.org; 2 Department of Bromatology, Faculty of Pharmacy, University of Belgrade, 11000 Belgrade, Serbia; nevena.ivanovic@pharmacy.bg.ac.rs (N.D.I.); brizitadjordjevic@pharmacy.bg.ac.rs (B.D.); 3 Department of Medical Biochemistry, Faculty of Pharmacy, University of Belgrade, 11000 Belgrade, Serbia; neda.milinkovic@pharmacy.bg.ac.rs; 4 Clinic for Endocrinology, Diabetes and Diseases of Metabolism, Clinical Center of Serbia, 11000 Belgrade, Serbia; snezanapolovina@gmail.com; 5 School of Medicine, University of Belgrade, Clinic for Endocrinology, Diabetes and Diseases of Metabolism, 11000 Belgrade, Serbia; msumaracdumanovic@gmail.com; 6 Department of Protein Engineering and Biochemistry, Institute of Virology, Vaccines and Sera “Torlak”, 11000 Belgrade, Serbia; minicrajna@gmail.com; 7 Department off Cell and Tissue Biology, Faculty of Biology, University of Belgrade, 11000 Belgrade, Serbia

**Keywords:** obesity, hematological parameters, *Lactiplantibacillus plantarum 299*v (DSM9843), *Saccharomyces cerevisiae* var. *boulardii*, octacosanol, clinical study

## Abstract

Background/Objectives: The prevalence of obesity worldwide has rapidly increased. Numerous studies showed a beneficial effect of probiotics in obese individuals, and changes in hematological parameters are observed in obesity. Therefore, the aim of this study was to investigate the effect of a novel probiotic approach on the red blood cells (RBCs) and platelets. Methods: Twenty-five obese women participated in a randomized, placebo-controlled study and were divided into the experimental group (one capsule daily containing *Lactiplantibacillus plantarum 299*v (DSM9843), *Saccharomyces cerevisiae* var. *boulardii*, and 40 mg octacosanol; *n* = 13) and the placebo group (*n* = 12). Blood samples were collected for light microscopic examination, morphometric analysis, and an automated hematology analyzer. A possible relationship between hematological parameters and body mass index (BMI), a common indicator of obesity, was investigated using Spearman correlation. The plasma concentration of soluble P-selectin and fibrinogen were determined using an ELISA assay. All measurements were performed before (T0) and after 12 weeks of supplementation (T1). Results: The three-month supplementation of probiotics improved hemoglobin levels, chromic status, and red blood cell morphology. The mean platelet volume (MPV), a measure of platelet size, was restored to normal levels, platelet morphology was improved, and the number of activated platelets was significantly reduced (*p* < 0.05). A strong negative correlation (r = −0.5904, *p* < 0.05) was found between BMI and platelet distribution width (PDW), a measure of variation in platelet size and shape. Conclusions: The results show that the probiotic approach improves morphology and normalizes the values of disturbed hematological parameters of RBCs and platelets in obese women.

## 1. Introduction

Obesity is a complex, multifactorial disease characterized by excessive fat accumulation in the body. As a state of low-grade inflammation, it increases the risk of numerous comorbidities, such as type 2 diabetes, hypertension, myocardial infarction, and stroke [1]. Excessive fat accumulation, especially visceral obesity, is associated with increased levels of proinflammatory cytokines, markers of oxidative stress, elevated circulating C-reactive protein (CRP), and serum leptin levels [2,3,4]. Under such conditions, cells, especially blood cells, are constantly exposed to an altered microenvironment, and their structural and functional properties can change. Obesity is often associated with an increased total white blood cell (WBC) count [5]. Several articles have described changes in red blood cells (RBCs) and platelet indices, such as changes in RBCs shape, volume, and size [6]; increased RBCs aggregation [7]; changes in hematological parameters indicative of platelet activation; and an increase in fibrinogen- and platelet-derived soluble P-selectin (sP-selectin) [8,9]. Among the platelet indices, the mean platelet volume (MPV) is increased in participants with a higher body mass index (BMI), a common indicator of obesity [10]. These and other studies suggest that hematological parameters may be a useful tool for tracking the progression of obesity and associated comorbidities but also point to the need for new therapeutic strategies.

It is well known that the composition of the gut microbiota in obese populations differs from that of lean, healthy individuals, and its alterations are closely associated with the development of obesity [11]. The beneficial effects of probiotics on energy, glucose, and lipid metabolism are already recognized [12], as is their ability to reduce abdominal obesity [13,14,15]. Our recent studies have investigated the valuable effects of a novel probiotic approach. For example, we have shown that the novel probiotic approach positively influences the expression of certain miRNAs and mRNAs involved in the regulation of inflammation and adipogenesis [16] and decreases concentrations of CRP and interleukin-6 (IL-6) in obese women [17]. However, as far as we are aware, the effect of a probiotic formulation on blood indices in humans has not yet been investigated.

Given the growing need for dietary supplements and nutraceuticals for weight loss, the aim of this study was to investigate erythrocyte and platelet indices and their potential changes in obese women after a novel probiotic approach. Due to their well-characterized anti-inflammatory role and trophic effect in the gastrointestinal tract, *Lactiplantibacillus plantarum 299*v [18,19,20] and *Saccharomyces cerevisiae* var. *boulardii* [21] have been combined with policosanol, a nutraceutical that reduces platelet aggregation [22,23].

## 2. Materials and Methods

### 2.1. Study Design, Subjects and Eligibility Requirements

Thirty-two obese (BMI ≥ 30 kg/m^2^) women of reproductive age (29–50 years) were recruited for this randomized, double blind, placebo-controlled, parallel study. The study was approved by the Ethics Committee of the Clinical Centre of Serbia (number 31/28, dated 21 February 2019) and registered in the Australian New Zealand Clinical Trials Registry (ACTRN12622000696796). The start of recruitment period was 10 June 2020, and the end of recruitment period was 6 July 2022. All participants were recruited from the Belgrade area and within the Clinical Centre of Serbia subject database via advertisements, phone calls, and word of mouth by researchers. Exclusion criteria were patients younger than 18 years; menopausal women; patients with chronic kidney, thyroid, and gastrointestinal disease; patients with anemia; patients with previous oncological disease; patients who had received probiotic, prebiotic, or antibiotic therapy in the last month; patients with hypersensitivity/intolerance to any of the product’s ingredients; and patients undergoing pregnancy or breastfeeding and who had not given birth within the last year. Written informed consent was obtained from participants prior to the start of the study, and the study was conducted in accordance with the Declaration of Helsinki.

**Figure 1:**
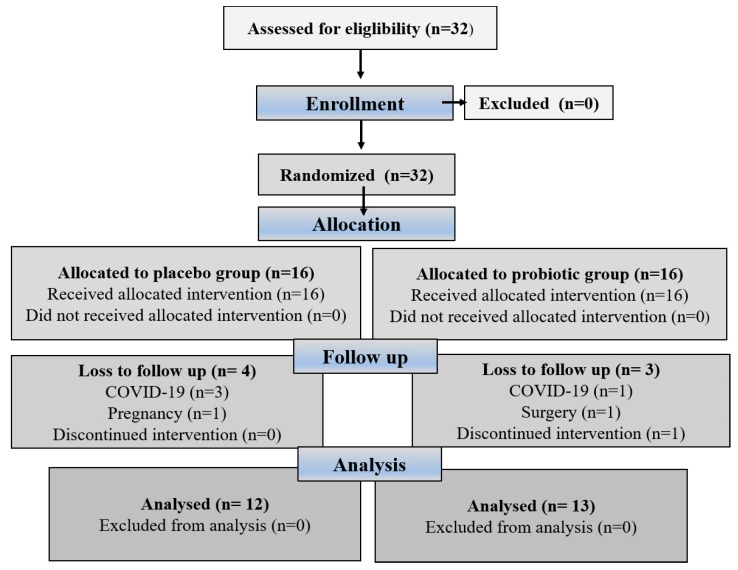
The CONSORT flowchart shows the process of participant recruitment and allocation.

Eligible participants were assigned a unique randomization number by a blinded medical supervisor, who entered the participants and assigned them to the interventions. The method used to create the randomization sequence for assigning subjects to the different groups was simple randomization using a randomization table created with computer software, and the random assignment was masked by sequentially numbered bins. Researchers, participants, outcome assessors, and practitioners were blinded to treatment allocation. Neither the participants nor the investigators knew which treatment they would receive in this study because the manufacturer packaged the supplements and placebos in plain boxes with the study code “1” or “2”; they were the same in terms of color, size, and shape to disguise the order until the intervention was assigned. Compliance with supplementation was verified by counting the pills. Adherence to supplementation was greater than 90% for all participants who completed the study. Figure 1 illustrates the participant recruitment and allocation process. Participants were contacted by telephone once a week and asked about possible side effects. In addition, participants were given advice on healthy eating and lifestyle habits to follow during the intervention phase. We hypothesized that probiotic supplementation would improve hematological indices and fibrinogen and sP-selectin levels (primary outcomes) in obese women. Secondary outcome aimed to show positive correlation between improved hematological indices and lower BMI.

### 2.2. Nutritional Supplementation

Sixteen patients received one capsule of placebo (control group—maltodextrin), and sixteen participants received one capsule of probiotics (7 × 10^10^ CFU *Lactiplantibacillus plantarum 299*v (DSM9843), 5 × 10^9^ CFU *Saccharomyces cerevisiae* var. *boulardii*, and 40 mg octacosanol) daily for three months and were asked to take one capsule daily after breakfast. Octacosanol originate from sugarcane, and dry extract of sugarcane (*Saccharum officinarum*) wax used in our study was standardized to 90% octacosanol (40 mg octacosanol, AbelaPharm Ltd., Belgrade, Serbia). Blood samples were taken early in the morning after a 12 h overnight fast at two time points: the day before the start of the intervention, defined as period 0 (T0), and after 90 days of oral supplementation, defined as (T1), for both the placebo and experimental groups. BMI was determined by trained personnel using a Tanita BC-401 electronic scale (Tanita, Amsterdam, the Netherlands) at the Clinical Centre of Serbia.

### 2.3. Laboratory Tests of the Hematological Parameters

Blood samples were collected in Vacutainer tubes containing EDTA disodium (anticoagulant). The plasma was immediately separated by centrifugation, and the aliquots were frozen at −80 °C for ELISA analysis. Hematological analysis was performed at the Clinical Center of Serbia using the XN-1000 automated blood analyzer (Sysmex, Manchester, UK). Evaluated parameters included red blood cell indices (red blood cell count—RBCs, red blood cell distribution width—RDW, hemoglobin—Hgb, hematocrit—HCT, mean corpuscular volume—MCV, mean corpuscular hemoglobin—MCH, and mean corpuscular hemoglobin concentration—MCHC) and platelet indices (platelet count—PLT, mean platelet volume—MPV, platelet distribution width—PDW, and plateletcrit—PCT) [24].

### 2.4. The Light Microscopic Examination of RBCs and Platelets Structural Alterations

Two blood smears were taken from each subject to examine the morphology of the RBCs and platelets. For the cytological examination, blood smears were routinely prepared from capillary blood and dried, fixed, and stained with May-Grunwald–Giemsa stains [25]. For each group, 200 stained images were taken at 100× magnification to analyze the color and shape of the RBCs as well as the appearance of the platelet granules and the color and shape of the cytoplasm. Based on these morphological characteristics, platelets were classified into three groups: granular (most of the cytoplasm is deeply stained), hypogranular (with few or no granules), and activated platelets (roundish platelets with droplets and granules in the center of the platelets) [26]. The images were also used to determine the size of platelets, which was measured as the maximum Ferret’s diameter, the longest distance between any two points on the object, commonly used in light microscopy to measure the size of irregularly shaped particles [27]. All measurements were performed with Fiji, an open-source distribution of the ImageJ image analysis software, version 1.54 (NIH, Bethesda, MD, USA).

### 2.5. Determination of Fibrinogen and sP-Selectin Levels

Peripheral venous blood was collected after overnight fasting in vacutainers with ethylenediaminetetraacetic acid (EDTA) for determination of sP-selectin, and fibrinogen was determined in citrated plasma by the Clauss coagulometric method using the BFT II Analyzer coagulometer. The concentration of sP-selectin (CD62P) was determined using a commercial enzymatic immunoassay (human ELISA, EH3818, FineTest, Wuhan, China) according to the manufacturer’s instructions. In brief, 100 µL of the pre-diluted sample was added per well and incubated at 37 °C for 1.5 h, followed by two washes (Rayto RT-2600C automatic washing machine, Shenzhen, China) in the wash buffer. Then, 100 μL of biotin-labeled antibody was added per well and incubated at 37 °C for 1 h. The plate was washed again, and 100 μL of horseradish peroxidase (HRP)-streptavidin conjugate was added per well and incubated at 37 °C for 30 min. After extensive washing, 90 μL of 3,3′,5,5′-tetramethylbenzidine (TMB) substrate was added per well and incubated at 37 °C for 10 min. The reaction was stopped by adding 50 μL of stop solution per well, and the absorbance of the samples was measured at 450 nm in duplicate using a Rayto RT-6100 automated reader.

### 2.6. Statistical Analysis

Statistical analysis was performed with GraphPad Prism 10.4.2 software (GraphPad Software, San Diego, CA, USA). D’Agostino and Pearson’s normality test was used to test the data for normality. Student’s *t*-test was used to compare two time points (T0 and T1) under the same experimental conditions. The results are expressed as mean ± statistical error (SEM). In addition, the changes in the variables in both groups were expressed as a delta value in % (Δ), useful for comparing changes between two sets of numbers. The delta value was calculated using the following formula: ((T1/T0) − 1) × 100), where T0 is the average value of the variables before supplementation for a given group and T1 is the average value of the variables after supplementation for a given group. The correlation of hematological parameters with BMI was evaluated by Spearman correlation. The level of statistical significance between the variables was defined using the *p* < 0.05 criterion. Interpretation of the strength of the correlation coefficients was accepted according Prion and Haerling’s study [28].

## 3. Results

### 3.1. Hematological and Morphological Analysis of RBCs and Platelets

The hematological parameters of the subjects before and after supplementation (placebo or probiotic) are listed in Table 1. Two of the parameters examined were found to be outside the reference range. Namely, the mean MPV value before administration (T0) in the experimental group was above the reference values and returned to normal values at the end of probiotic supplementation. In addition, the mean PDW values were below the reference values in both groups and at both time points (T0 and T1). Probiotic supplementation increased mean PDW levels from T0, bringing them closer to the reference range. Compared to baseline (T0), the mean RDW value was increased significantly (*p* < 0.05) in T1 in the placebo group.

**Figure 2:**
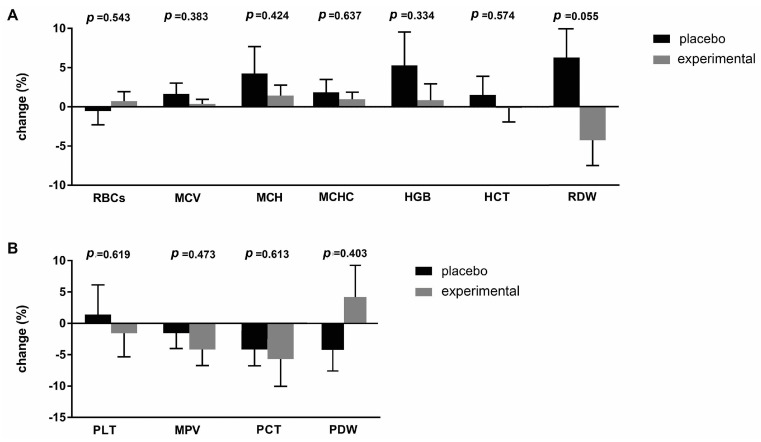
Percentage changes (Δ) in the mean values of (**A**) erythrocyte and (**B**) platelet parameters in the placebo and probiotic groups. The delta value was calculated using the following formula: ((T1/T0) − 1) × 100. The bars show the mean ± SEM. Statistical significance was set at *p* < 0.05.

To better understand the differences between the placebo and probiotic groups after supplementation, the delta value (Δ) of the hematological parameters was calculated and expressed as a percentage change (Figure 2). The greatest change was observed in the RDW parameter (−10.56 ± 5.11; *p* = 0.055), which almost reached statistical differences after three months of probiotic administration (Figure 2A). Figure 2B shows the percentage changes in platelet parameters. The largest difference was observed for the PDW parameter (−5.110 ± 5.98), which, however, did not reach a statistically significant difference.

**Figure 3:**
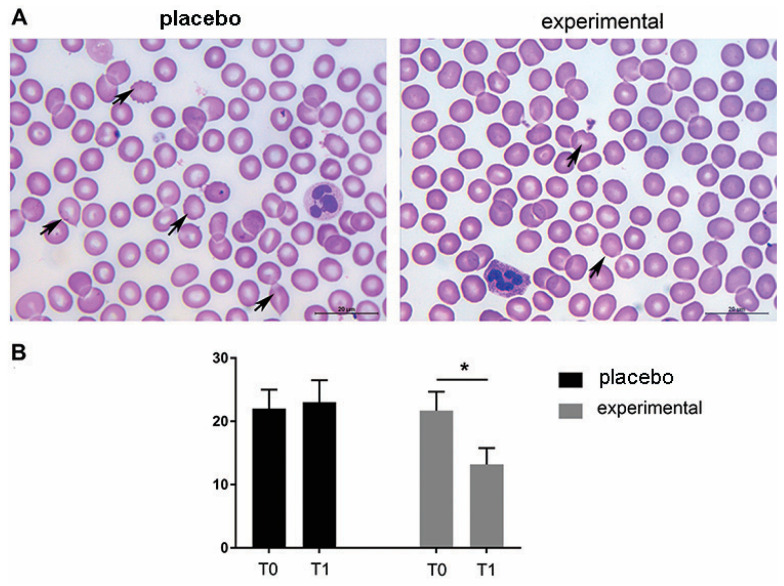
Representative light micrographs of blood smears show that the morphological abnormalities of erythrocytes decreased after three months of probiotic supplementation. (**A**) May-Grunwald–Giemsa staining of blood smears. Abnormally shaped RBCs are indicated by arrows. Scale bar: 20 µm. (**B**) The quantification of echinocytes and dacrocytes as a percentage of all erythrocytes. The bars show the mean ± SEM. Statistical significance was set at *p* < 0.05, (*).

The morphological analysis of the blood smears of both the placebo and the experimental group at T0 showed the presence of erythrocytes of different sizes and hypochromic erythrocytes. In addition, changes in erythrocyte shape were frequently observed at T0. Thus, moderate rouleaux formation was observed at T0 in both groups, and the presence of echinocytes and dacrocytes was frequently observed (Figure 3A). In contrast to the placebo group (T0 and T1, respectively) and the experimental group (T0), a decrease in morphologic abnormalities in size, shape, and staining properties of the RBCs was observed after three months of probiotic administration (probiotic group—T1). These microscopic observations were confirmed by the routine cell counting method (Figure 3B).

**Figure 4:**
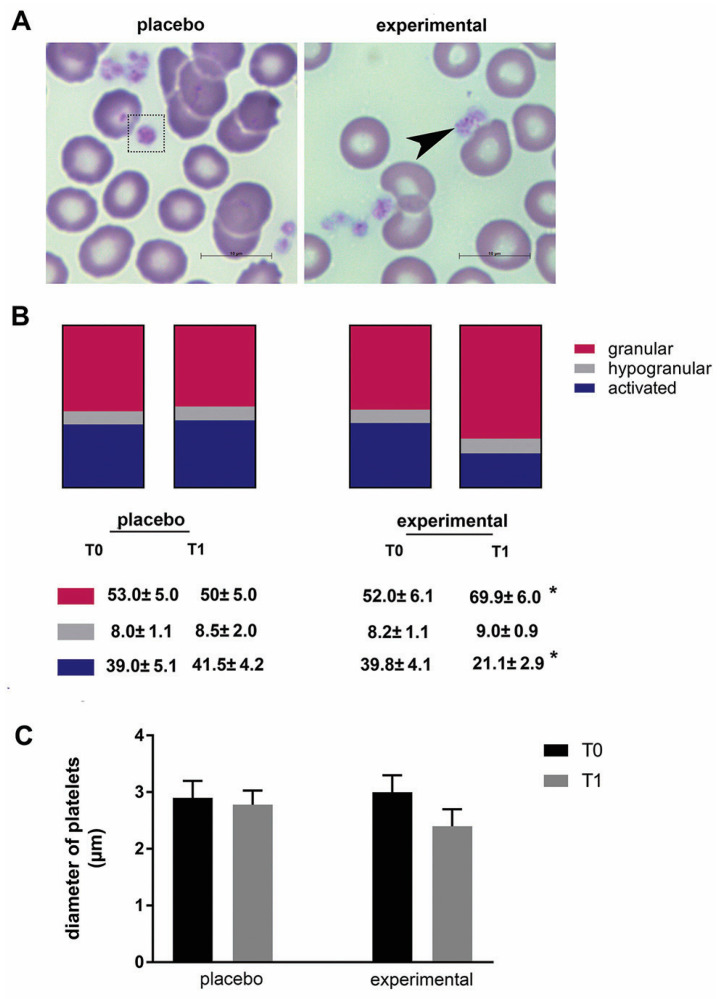
The morphology of platelets and the morphometric analysis. (**A**) Representative microscopic images of May-Grunwald and Giemsa staining in the placebo and probiotic groups after 3 months of supplementation. Scale bar: 10 µm. The dashed square indicates a large platelet, and the arrow indicates the presence of typical granular platelets. (**B**) The percentage of granular, hypogranular, and activated platelets. (**C**) The diameter of the blood platelets. Values are given as mean ± SEM. Statistical significance was set at *p* < 0.05, (*). Comparison with the corresponding T0 group.

The morphological changes of platelets were then examined (Figure 4A). In general, platelets of all samples differed in size, shape, and granular appearance. Based on their morphological characteristics, granular, hypogranular, and activated platelets were distinguished. These morphological observations were further analyzed using stereology, and a statistically significant change was found in the probiotic group. Compared to baseline (T0), the proportion of granular platelets increased significantly (*p* < 0.05), while the proportion of activated platelets decreased significantly after three months of probiotics (*p* < 0.05) (Figure 4B). In addition, there was a decreasing trend in platelet diameter after three months of probiotic administration compared to the corresponding control (T0), but this did not reach a statistical difference (Figure 4C).

### 3.2. Analysis of Fibrinogen and sP-Selectin Changes

**Figure 5:**
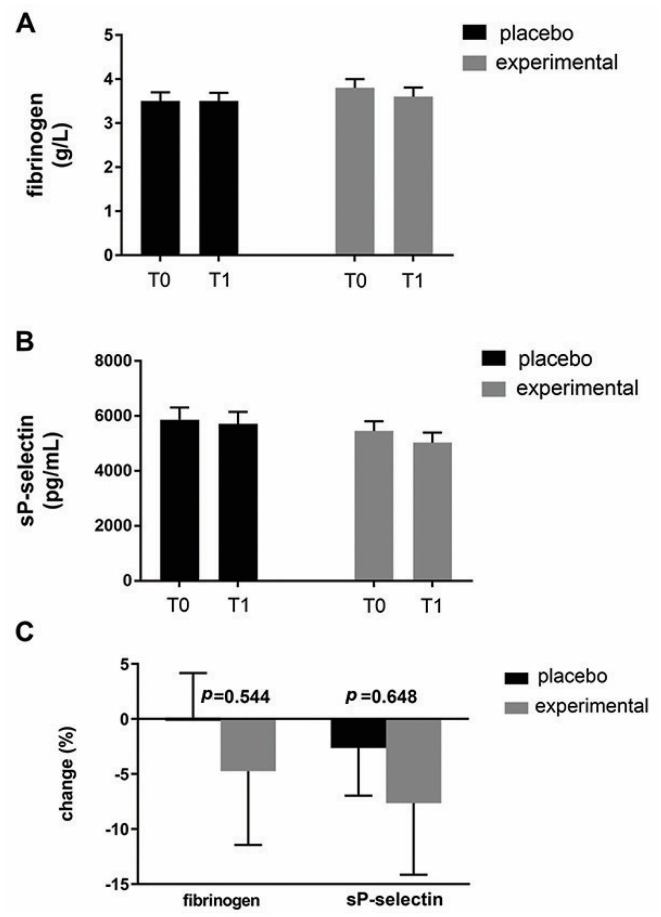
An analysis of changes in fibrinogen and sP-selectin. (**A**) Fibrinogen and (**B**) sP-selectin levels in the blood of the placebo and probiotic groups. (**C**) Percentage changes in the mean values of fibrinogen and sP-selectin in the placebo and probiotic groups. The delta value (Δ) was calculated according to the following formula: ((T1/T0) − 1) × 100. The bars show the mean ± SEM. Statistical significance was set at *p* < 0.05.

Figure 5 shows the mean fibrinogen level (Figure 5A) and the mean sP-selectin level (Figure 5B) in both groups. As can be seen, probiotic supplementation did not result in significant changes in these parameters within or between groups. Expressed as a percentage change (Figure 5C), there was a decreasing trend for both parameters in the probiotic group compared to the placebo group. Compared to fibrinogen (−4.86 ± 7.81), a greater change was observed for sP-selectin (−5.05 ± 9.98).

### 3.3. Correlation Between BMI and Hematological Parameters

**Figure 6:**
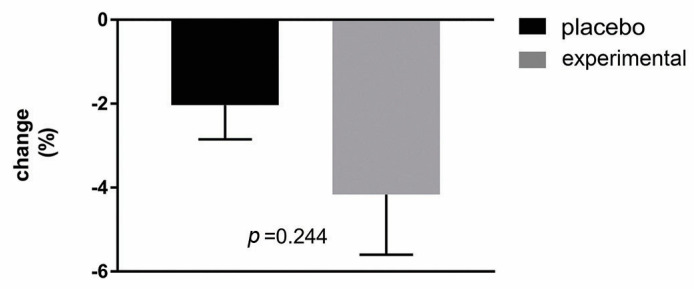
Percentage changes in BMI in the placebo and probiotic groups. The delta value (Δ) was calculated using the following formula: ((T1/T0) − 1) × 100. The bars show the mean ± SEM. The statistical significance was set at *p* < 0.05.

In order to analyze possible correlations, the change in BMI was first determined (Figure 6). As can be seen, the analysis showed a decreasing trend in BMI change after three months of probiotic administration compared to the placebo group but without a statistically significant difference.

The Spearman correlation coefficients of the delta values between BMI and blood parameters were then analyzed (Table 2). A statistically significant negative correlation was observed between ΔBMI and ΔPDW in the probiotic group (r = −0.5904, *p* = 0.049). In addition, a moderate negative correlation was observed between ΔBMI and ΔMCH (r = −0.452, *p* = 0.067) and ΔBMI and ΔMCHC (r = −0.522, *p* = 0.071). Thus, the participants who had a lower BMI after probiotic supplementation had a higher PDW, and the PDW was closer to the normal reference value. Although not statistically significant, a similar trend was observed for the hemoglobin parameters MCH and MCHC.

## 4. Discussion

Previous studies have shown that an increase in body mass leads to changes in numerous biochemical and blood cell parameters [2,3] and that gut microbiota in obese populations differs from that of lean, healthy individuals [5,6,11]. According to the literature, there are no studies that have targeted the influence of probiotic supplementation on hematological indices in obese individuals. Here, we showed that probiotic supplementation reduces morphological abnormalities and improves hematological parameters disturbed in obese women. Those changes are discussed below.

The results of the present study that show the increased MPV, a general indicator of platelet activity, are consistent with other reports in obese participants who showed that an increased MPV is strongly associated with abdominal obesity and waist circumference [29,30]. Here, we found that probiotic supplementation restored MPV values to normal range in the experimental group. In addition, we found that PDW values, a quantitative measure of platelet anisocytosis (variations in size and shape), were below the normal PDW range in the obese subjects. The mechanism underlying the increased MPV and decreased PDW in obese participants is not well understood. Taking into account that obesity is considered as a state of low-grade inflammation, a possible explanation could be related to the inflammatory state [31,32] and the increased utilization of large, activated platelets [33]. Further, restoration of the MPV parameter is accompanied by the improvement of PDW values, supporting our hypothesis about the effects of probiotics on platelets’ phenotype. Moreover, when the values were expressed as a delta value (Δ), we showed that percentage changes were most pronounced for the RDW parameter, a quantitative measurement of RBCs anisocytosis (variations in size and shape), which almost reached a significant level, followed by the aforementioned platelet parameter—PDW, both in the probiotic group.

A morphological analysis of the blood smears was then carried out to gain a better understanding of the structural changes in the erythrocytes and platelets. The morphological observations were combined with morphometric analysis and stereology, and a decrease in the morphological abnormalities of RBCs and platelets was observed after probiotic administration. Overall, the analysis of the blood smears showed that supplementation with probiotics had beneficial effects on three levels. Thus, (i) the size and shape of the erythrocytes were brought back to a similar general size and shape by the probiotic treatment. These changes were followed by (ii) an increased incidence of normochromic red blood cells. Finally, (iii) probiotic supplementation increased the proportion of discoid-shaped and granular platelets, one of the most important morphological features of unstimulated platelets [34]. Together with the automated hematology data analyzer, all these results undoubtedly confirm the beneficial effects of probiotics on the changes in erythrocyte and platelet size and shape as well as on the chromic status of erythrocytes. A similar trend in most of the examined parameters observed in our preliminary study was presented, which included a smaller group of participants.

In addition to platelet activation, obesity can be considered a prothrombotic state and could be associated with increased fibrinogen and sP-selectin levels [2,35]. Here, we did not observe an increase in fibrinogen and sP-selectin in obese women. Expressed as percentage change, there was only a decreasing trend for both parameters in the probiotic group compared to the placebo group. A possible explanation, at least for sP-selectin, could be the uniform lipid profile in our participants who participate in the same clinical trial since it has been shown that sP-selectin correlates with triglycerides in obese patients [16,35].

Finally, Spearman correlation coefficients of the delta values between BMI and blood parameters were then analyzed to determine the relationship between the variables. Despite the fact that probiotics can cause weight loss, only a tendency to reduce BMI was observed in our study. However, the correlation analysis between the changes in hematological parameters and BMI gave us additional insight into the important effect of probiotics in obese women. The Spearman correlation analysis revealed a significant negative correlation between the changes in BMI and PDW in the probiotic group. This additionally supports our findings that probiotics positively affect platelet morphologic uniformity and suggests that participants with a lower BMI had more uniform platelets. In addition, a moderate, negative correlation between BMI and hemoglobin content and concentration parameters (ΔBMI and ΔMCH and ΔBMI and ΔMCHC, respectively) almost reached significance in the probiotic group. As can be read in the literature, the relationship between hemoglobin levels in overweight/obese individuals remains controversial, depending on conditions such as age and gender [6,36,37]. The positive effect of probiotics on erythrocyte chromic status we observed here may be a consequence of the previously described beneficial effects of *L. plantarum 299*v products on hemoglobin and iron levels, which are due to increased intestinal iron absorption [38]. It is also worth highlighting that products of *Lactiplantibacillus plantarum 299*v have a significant antioxidant capacity and the ability to reduce cytokines, thereby reducing systemic inflammation [39]. In line with this are our recently reported data that the same probiotic approach reduced tumor necrosis factor α (*TNF-alpha*) gene expression and levels of CRP and IL-6 in obese women [16,17]. Also, it has been shown that bioactive compounds of *Saccharomyces cerevisiae* var. *boulardii* show antioxidative capacity [40,41], while octacosanol exerts positive effects on platelet aggregation [42]. Considering that this is the first evaluation of the effects of probiotics on hematological indices in obesity, the exact mechanism of their action is unknown. Based on our previous findings and the results of other studies, the improvement in hematological indices shown in this study could be due to an improvement in inflammatory and antioxidant defense status. However, the evaluation of hematological parameters in conjunction with peripheral blood smears contributes to a better understanding of morphological abnormalities of erythrocytes and platelets in obesity and had a significant impact on the interpretation of laboratory results. According to our study, a novel probiotic formulation improves hematological and morphologic indices of erythrocytes and platelets in obese women. However, further studies are needed to better understand the effects of probiotics on hematological parameters in obese women, and the limitations of this study should be noted, particularly the small sample size. Although our intention was to test the physiological effects of the entire multicomponent formulation, reflecting the real-world use of such supplements, studies with mono-intervention arms will be essential to further decipher the individual and synergistic mechanisms of action.

## 5. Conclusions

Our results are the first study on the effects of probiotic supplementation on hematological parameters in obese women and underline the important role of probiotics in obesity. We showed that the probiotic formulation used here normalized MPV values and improved both PDW and RDW values, hemoglobin-related parameters, and morphologic indices of erythrocytes and platelets in obese women. Our findings provide important information for the potential use of probiotics to ameliorate obesity-related complications and the potential use of hematological parameters as simple and inexpensive indices in clinical practice to assess the risk of obesity-related disease.

However, before probiotics can be used to treat obesity, their role should be investigated in a larger number of patients of both genders. It is also important to determine the effective dose and maximum duration of use of probiotics.

## Figures and Tables

**Table 1:** The effect of 3 months probiotic supplementation on the hematological indices of red blood cells and platelets in obese women. The evaluated parameters included erythrocytes and platelet indices. The day before the start of the intervention was defined as period 0 (T0), and T1 defined the end of the intervention for both the placebo and experimental groups. Data are presented as mean ± SEM. Statistical significance was set at *p* < 0.05, (*).

Parameter	Referent Values	Placebo		*p* Value	Experimental		*p* Value
		T0	T1		T0	T1	
RBC (×10^12^L)	3.86–5.08	4.77 ± 0.11	4.73 ± 0.01	0.709	4.59 ± 0.06	4.63 ± 0.07	0.671
HGB (g/L)	122–157	144.3 ± 11.4	150.4 ± 12.6	0.724	132.8 ± 3.1	133.6 ± 3.0	0.855
HCT (L/L)	0.356–0.47	0.4101 ± 0.007	0.4153 ± 0.007	0.990	0.4083 ± 0.005	0.4072 ± 0.008	0.910
MCV (fL)	83–97	85.5 ± 1.2	88.1 ± 1.1	0.123	88.9 ± 1.2	89.2 ± 1.4	0.873
MCH (pg)	27.4–33.9	28.1 ± 1.0	29.0 ± 0.5	0.358	28.8 ± 0.6	29.2 ± 0.5	0.611
MCHC (g/L)	320–360	324 ± 5.0	331 ± 3.0	0.234	325 ± 3.0	328 ± 3.0	0.487
RDW (%)	12–15.5	13.7± 0.3	14.8 ± 0.4	0.040 *	14.9 ± 0.7	14.0 ± 0.4	0.905
PLT (×10^9^L)	150–450	293.1 ± 13.4	294.2 ± 17.0	0.960	268.9 ± 11.7	262.1 ± 11.1	0.677
MPV (fL)	6.8–10.4	9.78 ± 0.41	9.76 ± 0.39	0.972	10.52 ± 0.37	10.08 ± 0.40	0.430
PCT (%)	0.158–0.425	0.294 ± 0.02	0.281 ± 0.01	0.557	0.285 ± 0.02	0.268 ± 0.01	0.444
PDW (%)	14–17.8	13.17 ± 0.4	13.02 ± 0.8	0.872	13.32± 0.5	13.75 ± 0.8	0.659

**Table 2:** Spearman correlation coefficients of delta values between BMI and hematological parameters. Erythrocytes indices (number of erythrocytes—RBCs, red blood cell distribution width—RDW, hemoglobin—Hgb, hematocrit—HCT, mean corpuscular volume—MCV, mean corpuscular hemoglobin—MCH, and mean corpuscular hemoglobin concentration—MCHC) and platelet indices (platelet count—PLT, mean platelet volume—MPV, platelet distribution width—PDW, and plateletcrit—PCT). The correlation coefficient (r), the significance level (*p*), and the number of patients (*n*) are shown. The strength of correlation coefficients: 0.00 to 0.20, negligible; 0.21 to 0.40, weak; 0.41 to 0.60, moderate; 0.61 to 0.80, strong; 0.81 to 1.00, very strong). Statistical significance was set at *p* < 0.05, (*).

	Placebo *n* = 12		Experimental *n* = 13	
Variable	r	*p* Value	R	*p* Value
**RBC** (×10^12^L)	0.259	0.438	0.031	0.919
**Hgb** (g/L)	−0.067	0.837	−0.379	0.202
**HCT** (L/L)	0.059	0.865	−0.236	0.437
**MCV** (fL)	−0.358	0.251	−0.357	0.232
**MCH** (pg)	0.023	0.951	**−0.528**	**0.067**
**MCHC** (g/L)	0.232	0.489	**−0.522**	**0.071**
**RDW** (%)	0.001	0.973	0.250	0.521
**PLT** (×10^9^L)	0.210	0.533	0.442	0.116
**MPV** (fL)	−0.133	0.678	−0.147	0.646
**PCT** (%)	0.238	0.582	0.349	0.241
**PDW** (%)	0.232	0.489	**−0.591**	0.049 *
**Fibrinogen** (g/L)	0.071	0.906	0.200	0.613
**P-selectin** (pg/mL)	0.025	0.958	0.035	0.890

## Data Availability

Data are unavailable due to ethical restrictions.

## Abbreviations

The following abbreviations are used in this manuscript:

CRP	C-reactive protein
BMI	body mass index
RBCs	red blood cell count
RDW	red blood cell distribution width
Hgb	Hemoglobin
HCT	Hematocrit
MCV	mean corpuscular volume
MCH	mean corpuscular hemoglobin
MCHC	mean corpuscular hemoglobin concentration
PLT	platelet count
MPV	mean platelet volume
PDW	platelet distribution width
PCT	Plateletcrit
TNFα	tumor necrosis factor alpha
IL-6	Interleukin 6
